# Making Coal-Fires

**Published:** 1861-01

**Authors:** 


**MAKING COAL-FIRES.**—Good hard coal is in square lumps, and breaks with a smooth, shining fracture. Bad coal has flat pieces of a dull color, as thick as the palm of the hand, and of greater or less size, which when burnt remain hard, heavy, and become whitish—hence called "bone." If a common scuttle-ful of coal, about twenty-five pounds, yields, after the cinders are washed next morning, half a pound of white pieces, it is not good coal.

The kindling-wood should not be over four inches long, and should not be spread out over the paper or the shavings which are to kindle it, but should be moderately compact, so as to concentrate the heat given out to as few pieces of coal as possible, which pieces should not be larger than a walnut, nor much smaller, because a piece of coal must be heated through and through before it "catches fire;" hence, if the piece is large, the amount of wood used may not contain enough caloric to properly heat the coal, and the fire will go out, to the great discouragement of the servant, and the unreasonable wrathfulness of the served, making perhaps a "bad beginning," which is to cloud the whole day, impairing digestion, and exciting an ugly temper, the ungenial effects of which a whole family may be made to feel for the next twenty-four hours.

The wood may be covered, just out of sight, with walnut-sized pieces of coal, and when these have become red, cover them over with larger pieces, to be added to thereafter, as may be necessary.

If a coal-fire seems likely to go out, the most effectual way to complete the process is to riddle out the ashes, or to add more coal; for the ashes retain some heat to send up to small pieces; but if larger or too numerous, there is not enough caloric to heat them through and through, which must precede their enkindling. Sometimes a coal-fire, where the coal is fresh and is almost kindled, is thoroughly lighted up by introducing between or just under the pieces, a few splinters of lighted wood in such a manner as to disturb the coal as little as possible.

